# A randomized controlled trial to assess whether a telehealth-based contingency management intervention reduces alcohol use for individuals with alcohol use disorder

**DOI:** 10.1016/j.cct.2025.107807

**Published:** 2025-01-15

**Authors:** Julianne D. Jett, Diana Tyutyunnyk, Rachael Beck, Katharine Palmer, Danielle Ryan, Jesus Sanchez, Douglas L. Weeks, Sterling M. McPherson, Naomi Chaytor, Brian Kiluk, Martin A. Javors, Brett C. Ginsburg, Sean Murphy, Nathalie Hill-Kapturczak, Michael G. McDonell

**Affiliations:** a Department of Community and Behavioral Health, Elson S. Floyd College of Medicine, Washington State University, Spokane, WA, USA; b Department of Psychiatry and Behavioral Sciences, University of Texas Health San Antonio, TX, USA; c Department of Population Health Sciences, Weill Cornell Medicine, New York, NY, USA; d Department of Psychiatry, Yale School of Medicine, New Haven, CT, USA

**Keywords:** Telehealth, Contingency management (CM), Alcohol use disorder (AUD), Urine ethyl glucuronide (uEtG), Phosphatidylethanol (PEth), Addictions neuroclinical assessment framework

## Abstract

**Background:**

Contingency management (CM) is an intervention for alcohol use disorder (AUD) that reinforces abstinence, as confirmed by alcohol biomarkers. CM is usually brief (12–16 weeks) despite evidence that longer interventions have better long-term outcomes. Most CM models are in-person which can also be a barrier for treatment. Studies of longer duration telehealth-based CM models are needed.

**Aims:**

To determine if a telehealth-based CM model that utilizes phosphatidylethanol (PEth) to confirm abstinence is effective at reducing alcohol use during a 26-week intervention and 12-month follow-up. We will evaluate the impact of CM on alcohol-related outcomes, determine if Addiction Neuroclinical Assessment variables are associated with outcomes in follow-up, and whether savings related to decreased alcohol use offset intervention costs.

**Methods:**

Adults with AUD residing in the United States will be recruited via online advertising. Research procedures will be conducted virtually. Participants who submit a PEth-positive blood sample (≥20 ng/mL) at enrollment will be randomized to 26 weeks of either 1) online cognitive behavior therapy (CBT4CBT) with rewards not contingent on PEth results (Control group) or 2) CBT4CBT with a maximum of $1,820 of rewards contingent on PEth results (CM group). Efficacy outcomes of PEth-negative tests (primary) and PEth-defined excessive drinking (≥200 ng/mL; secondary) will be assessed. Predictors of intervention outcomes and economic viability will also be investigated.

**Discussion:**

If this telehealth-delivered PEth-based CM intervention reduces alcohol use and is cost-effective, it could be used to provide effective treatment to millions of individuals with AUD who do not receive in-person care.

## 1. Introduction

Despite the availability of treatment options for alcohol use disorder (AUD), few individuals with AUD seek treatment [1]. Poor treatment utilization may result from barriers associated with in-person care, e.g., inconvenience, transportation costs, stigma [2]. During the COVID-19 pandemic, 60% of substance use disorder patients received some portion of their substance-related treatment via telehealth [3]. Prior to the pandemic, virtual treatments were also evaluated and disseminated as treatment options for patients with substance use disorders. These included online cognitive behavioral therapies, such as Computer Based Training for Cognitive Behavioral Therapy (CBT4CBT) and telehealth-based contingency management (CM) [4,5]. However, this work has focused on telehealth treatment for illicit drug use, while telehealth treatments for AUD are limited to pilot studies [6,7]. Telehealth interventions have potential limitations (e.g., access to and comfort with technology), however its advantages likely outweigh disadvantages for individuals with AUD who do not or cannot access in-person care. Randomized controlled studies evaluating the efficacy of telehealth-based treatment models for AUD are needed.

CM is ideal for telehealth delivery given its simple procedural nature, which consists of individuals receiving tangible reinforcers in exchange for meeting treatment goals (e.g., reducing substance use, maintaining abstinence). Initial telehealth and app-based CM models indicate reductions in drug and alcohol use (e.g., DynamiCare, FDA-approved reSET) [6–12,23,26,27]. For AUD, these telehealth-based CM models have utilized transdermal monitors or repeated daily breath sampling, which can be costly, burdensome, and stigmatizing for patients [6,7,9,11]. Another factor that can impact the efficacy of a telehealth-based CM model is the intervention duration. A meta-analysis found that CM was associated with a 22% higher odds of post-treatment illicit drug abstinence in comparison to other interventions, and that the best predictor of long-term abstinence was the length of the CM intervention [33]. The utilization of alcohol biomarkers that have a longer window of detection is one method to extend telehealth-based CM duration while also reducing burden on patients through decreased sampling and visit frequency.

We previously conducted a pilot study for a telehealth-based CM model that used the blood biomarker phosphatidylethanol (PEth) to assess alcohol consumption [7]. The PEth homologue 16:0/18:1 can be detected for up to 28 days [8], thus assessing abstinence or low levels of drinking can occur as infrequently as once a month. Accordingly, our pilot study (*n* = 16) used a two-phase CM treatment approach. Phase 1 helped participants initiate abstinence with weekly PEth assessment and reinforcement of week-to-week decreases in PEth levels. Once PEth levels indicated sustained reductions in alcohol consumption (PEth <20 ng/mL), participants transitioned to Phase 2 where sampling and reinforcement decreased to once every two to four weeks. By reducing sample frequency, we were able to extend the CM intervention duration to 26 weeks. Participants reported high satisfaction with study procedures, to include self-collection of blood samples using the TASSO-M20 device, suggesting that our telehealth-based model was feasible. Additionally, CM participants were five times more likely than Control participants to submit PEth samples indicative of alcohol abstinence (*p* = 0.007), indicating that our telehealth-based CM model was also efficacious [7].

Based on these pilot results, we received funding to conduct a randomized controlled clinical trial of our telehealth-based CM intervention for AUD (R01AA031013; ClinicalTrials.gov ID NCT06265506). For this study, our primary aim is to determine whether the frequency of PEth-defined abstinence (<20 ng/mL) or absence of regular excessive drinking (<200 ng/mL) is higher in those receiving CM compared to the Control group during the intervention and follow-up periods. Additional aims include a) assessing group differences in alcohol-associated harms, (e.g., cigarette/tobacco use, drug use, costly medical care), b) describing the association between the Addictions Neuroclinical Assessment (ANA) framework variables (incentive salience, executive functioning, negative emotions) and alcohol outcomes [9], and c) conducting a cost-effectiveness analysis to determine the value of the CM group relative to the Control group, from the healthcare sector, state policymaker, and societal perspectives.

## 2. Methods

### 2.1. Study design

We aim to randomize 200 adults with an AUD from the contiguous United States. Targeted internet advertisements will recruit participants. Advertisements will direct individuals to the study landing page to complete a screening survey and provide contact information. Those potentially eligible will be scheduled for an enrollment visit via a Health Insurance Portability and Accountability Act compliant Zoom. During enrollment, an electronic consent form and eligibility interview will be completed. Additionally, a blood sample for the assessment of PEth will be self-collected using the TASSO-M20 device, along with a self-collected urine sample to assess the alcohol biomarker urine ethyl glucuronide (uEtG). Participants will ship their blood samples via prepaid envelopes for laboratory assessment and conduct uEtG testing on camera with staff using a dipcard.

**Fig. 1.**
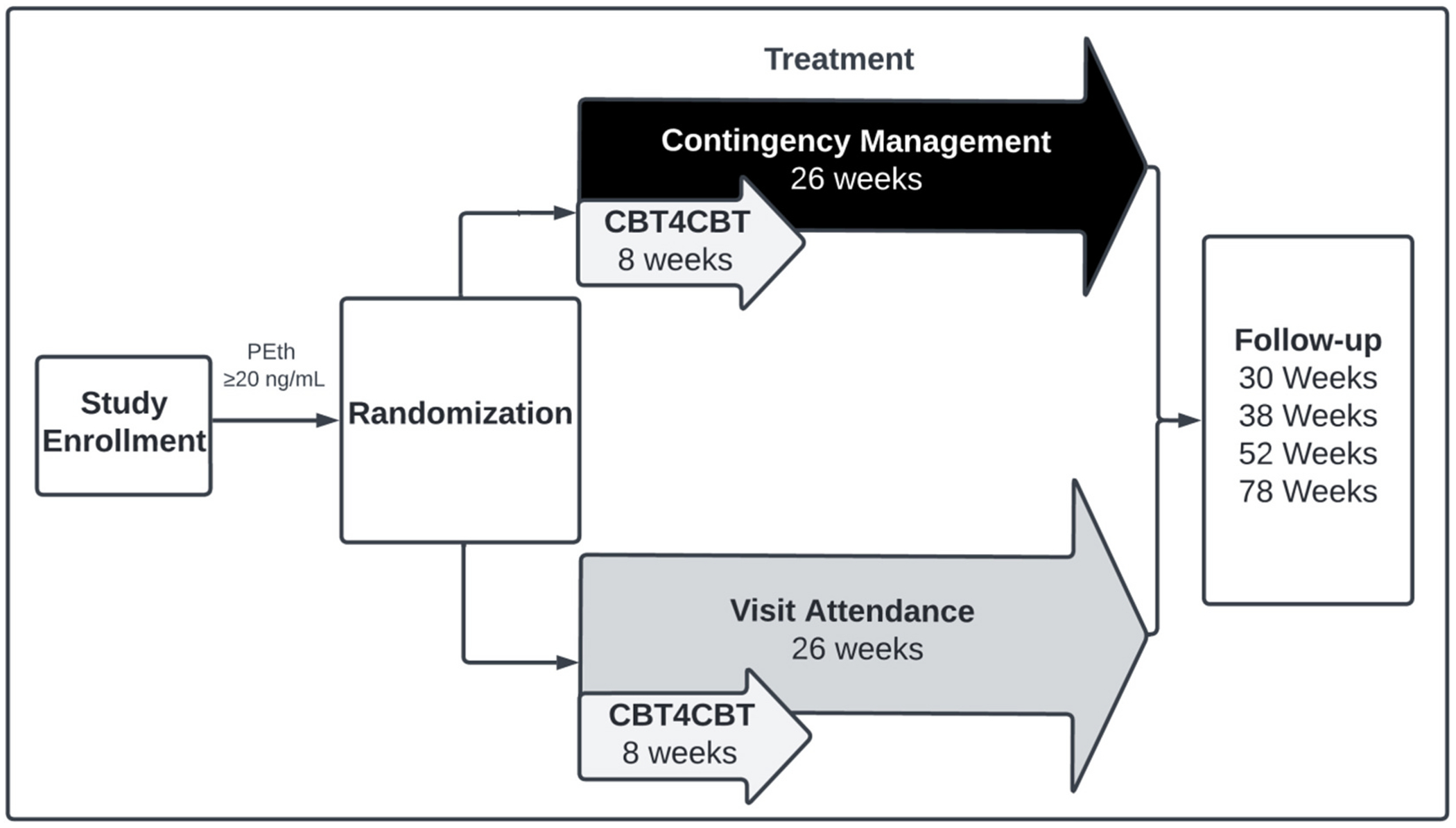
Study Flow: Following phone screening, participants will attend an enrollment visit via Zoom to further assess study eligibility and self-collect a blood sample for PEth analysis. If participants meet study eligibility, to include a PEth level that is ≥20 ng/mL, they will be randomized to either 26-weeks of the CM or Control group. After treatment, follow-up visits will be conducted over the span of 12 months (i.e., Weeks 30, 38, 52, 78).

Participants whose PEth levels are ≥20 ng/mL, indicating alcohol use in the last four weeks, will be randomized to a 26-week intervention period of either: 1) CM group, in which participants complete online CBT4CBT modules and receive reinforcers that are contingent on PEth results, or 2) Control group, in which participants complete online CBT4CBT modules and receive reinforcers regardless of PEth results. After the intervention period, participants will complete follow-up assessments at Weeks 30, 38, 52, and 78 (i.e., 12-month duration) to determine the long-term effects of CM and predictors of abstinence (see Fig. 1). All study visits will occur via Zoom with reinforcers delivered as electronic gift cards using Tango (e-gift cards; tango.com) with restrictions on the purchase of alcohol, tobacco, and firearms.

### 2.2. Study procedures

#### 2.2.1. Participant eligibility

Inclusion criteria include: 1) two self-reported heavy drinking episodes (>4 standard drinks for male assigned at birth, >3 standard drinks for female assigned at birth) or ≥14 standard drinks in the last 14 days at the time of screening; 2) a PEth level that is ≥20 ng/mL at study enrollment; 3) diagnosis of a current AUD as assessed by the Structured Clinical Interview for DSM-5 (SCID) [10]; 4) 18+ years of age; 5) residing in the contiguous USA; 6) fluent in English; 7) access to reliable internet for videoconferencing. Ineligibility criteria include: 1) current SCID diagnosis of a severe drug use disorder, excluding cannabis; 2) receiving other AUD treatment at study enrollment; 3) inability to provide informed consent based on the San Diego Brief Assessment of Capacity to Consent; 4) history of alcohol withdrawal-related seizures or alcohol-related hospitalization in the prior 12 months; or 5) psychiatrically or medically unsafe to participate, per the principal investigator. Should a participant enroll in another AUD treatment after randomization, they will remain in the study.

#### 2.2.2. Randomization procedures

Eligible participants will be assigned to study arms based on permuted block randomization in REDCap that is stratified by sex (male, female, other, prefer not to say) and PEth levels at the study enrollment visit (<200 ng/mL or ≥200 ng/mL). PEth ≥200 ng/mL equates to regular excessive drinking in the prior two to four weeks [11,12]. Participants will be informed of their treatment condition at the randomization visit, one week after enrollment (see Fig. 1).

### 2.3. Study interventions

#### 2.3.1. CBT4CBT

All randomized participants will receive CBT4CBT, which is an evidence-based computerized intervention for substance use that was adapted and tested for AUD [4,13–19]. CBT4CBT teaches the principles of cognitive behavioral therapy (CBT) through video, graphics, audio instruction, interactive exercises, and homework [20]. Participants will complete six modules during the first eight weeks of treatment (~40 min/module). The program tracks how long participants are logged in, the modules completed, homework assignments completed, and to what degree CBT principles are learned through quizzes. Participant engagement will be monitored and supported by staff during study visits.

#### 2.3.2. CM group

**Fig. 2.**
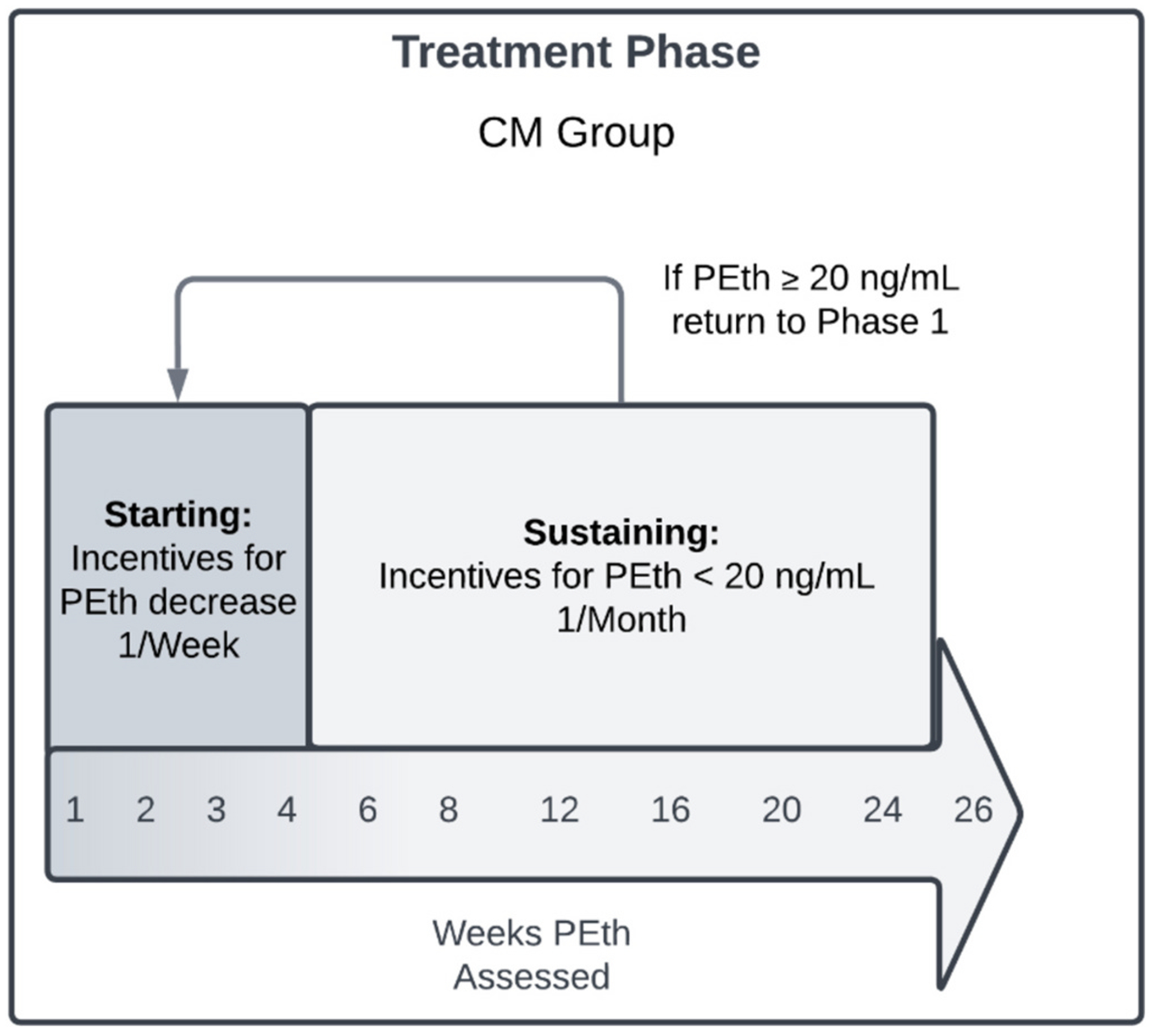
CM Treatment Schedule: CM participants will remain in the Starting Phase for the first four weeks of treatment or until their PEth levels indicate abstinence (<20 ng/mL). They will then transition to the Sustaining Phase of treatment, where they are reinforced for maintaining PEth levels below 20 ng/mL. Should a participant in the Sustaining Phase have a PEth level ≥20 ng/mL, they will return to weekly visits (i.e., Starting Phase). Once PEth levels are again <20 ng/mL, participants will return to monthly visits in the Sustaining Phase. Regardless of how many times a participant returns to the Starting Phase, the treatment duration will remain 26 weeks.

Participants will receive CBT4CBT in addition to their reinforcers being contingent on PEth results. The criteria for PEth-verified abstinence will differ depending on the treatment phase. During the Starting Phase, week-over-week decreases in PEth levels, regardless of the amount, will be reinforced until participants reach PEth <20 ng/mL, which is consistent with approximately four weeks of abstinence. After completing 4 weeks of the Starting Phase and/or achieving PEth <20 ng/mL, participants will transition to the Sustaining Phase with visits every two to four weeks, as long as abstinence is maintained. Should PEth levels indicate that drinking occurred since the last visit (≥20 ng/mL), CM participants will return to the Starting Phase with weekly visits until PEth levels once again indicate abstinence (see Fig. 2). Returning to the Starting Phase provides CM participants with additional support when a drinking episode occurs. Participants will continue the Sustaining Phase when their PEth levels return to abstinence (<20 ng/mL), and treatment duration will remain 26 weeks regardless of how long a participant remains in the Starting Phase or how many times they return to the Starting Phase.

During the Starting Phase, participants will receive $20 for the first PEth result that indicates reduced alcohol consumption (i.e., week-to-week decrease). They will then receive an additional $5 for every week of consecutive abstinence (i.e., week-to-week decrease during the Starting Phase, <20 ng/mL during the Sustaining Phase). PEth-positive samples (i.e., week-to-week PEth increase, PEth ≥20 ng/mL) will result in reinforcers being withheld until the next negative test, for which the participant will earn $20. A subsequent negative test will then reinstate the total reinforcers earned prior to testing positive, plus an additional $5. For example, if a participant previously earned $40 but then tests positive, they will receive $0 at the current visit. When the participant submits the next negative sample, they will earn $20, and then be eligible for reinstating their highest earned total (e.g., $40) plus an additional $5 when they have two consecutively negative samples. This reinforcer escalation schedule, reset, recovery approach is consistent with previous CM studies [21,22].

If CM participants have an unexcused missed visit or do not ship their sample within a week of the study visit, the PEth sample will be considered positive. In such cases, reinforcers will be withheld, and the participant will return to the Starting Phase. The maximum CM reinforcer that can be earned per week of abstinence is $90 (e.g., for visits occurring once every 4 weeks the max is $360). The maximum total amount that can be earned for negative PEth results during the CM intervention is $1,820.

#### 2.3.3. Control group

Participants will receive CBT4CBT with their reinforcers contingent on submitting blood samples, not PEth results. Blood sample collection will occur on a fixed schedule of once weekly (Weeks 1-4), then every other week (Weeks 6 and 8), followed by every four weeks (Weeks 12, 16, 20, and 24), and a last intervention visit occurring on Week 26 (see Fig. 1). Similar to previous studies, the amount of reinforcement allotted for each sample will be a base level of $20 or yoked to the CM group [23,24]. When Control participants submit a blood sample, they will receive a reinforcer amount that is equal to the average provided to CM participants during the previous month, resulting in earnings that approximate the CM group. For example, if the average earning per sample in the CM group was $50, Control participants will receive $50 for each sample submitted. Total Control group reinforcement could be as high as $1,820 but is expected to be 50% of that amount.

### 2.4. Data collection

Staff will collect data via Zoom at enrollment, randomization, treatment (Weeks 1–26), and follow-up visits (Weeks 30, 38, 52, 78). Measures that occur asynchronously will include the TestMyBrain cognitive tests and ANA questionnaire. The enrollment visit will take approximately three hours, while the randomization visit will take one hour. Weekly visits during the Starting Phase will average thirty minutes, while Sustaining Phase and follow-up visits will take an hour. Blood and urine samples will be collected at each visit in addition to the measures outlined in Table 1. Participants will receive $30 for the enrollment visit, $10 for the randomization visit, and $20 for each follow-up visit. To ensure timely submission of blood samples, all participants will receive a $10 bonus per sample if they ship the same day as their study visit. Compensation provided during the treatment phase is outlined in Section 2.3.

### 2.5. Measures

#### 2.5.1. PEth sample collection and analyses

Staff will mail study kits to participant residences. The kit will include a TASSO-M20 device for blood collection, isopropanol swabs, towelettes, a bandage, a urine cup, a uEtG dipcard, and a pre-paid shipping envelope. The TASSO-M20 device will be pre-labeled with the participant study ID and the visit number. Participants will self-collect blood from the upper arm with the TASSO-M20 device while videoconferencing with staff. Participants will press a button on the device to create a small lance in the skin, from which capillary blood will fill four plugs in the device (20 μL ± 5%/plug) over the span of five minutes (see tassoinc.com). Once completed, participants will remove the device, take the film cover over the plugs off to allow air drying, and seal the device in a prepaid FedEx envelope. Participants will mail the packet to the University of Texas Health San Antonio for PEth analysis. Our pilot study demonstrated that the TASSO-M20 device is a feasible method for collecting blood, e.g., consistency of blood volume collected, easy for participants to use, minimal pain [7].

PEth 16:0/18:1 levels will be assessed using a modified method for high-performance liquid chromatography with tandem mass spectrometry [25,26]. All reagents will be purchased from Thermo Fisher Scientific and Sigma Chemical (Fisher Scientific, Waltham, MA). PEth 16:0/18:1 analytical standard will be purchased from Cerilliant (Round Rock, TX). Deuterated PEth (16,0/18,1-*d*5, Echelon Biosciences, Salt Lake, UT) will serve as the internal standard. PEth calibrator (0–2000 ng/mL) and control samples will have a volume of 40 μL. Two plugs from each TASSO-M20 device will be analyzed. Each sample, excluding the blank control, will be spiked with 10 μL of the 0.1 μg/mL deuterated PEth, final concentration 25 ng/mL. PEth will be extracted from the calibrator, control, and experimental samples by the addition of isopropanol (1 mL) with vortexing (1 min), shaking at high speed (45 min, Eberbach Shaker), sonication using 5 second bursts (Q-Sonica, 10% max), and centrifugation at 3,200 g at 4°C (30 min). The clear supernatants will be transferred to new tubes and evaporated to residue with a gentle stream of nitrogen at 30 °C. The residues will be dissolved in 100 μL of a 1:1 resuspension solution of mobile phase A (40,60, 2 mM ammonium acetate,acetonitrile) and mobile phase B (isopropanol) and then transferred to 1.5 mL snap-cap polypropylene microcentrifuge tubes and centrifuged for 10 min (3,200 *g*) at 4°C. The eluted samples will be transferred to 300 μL polypropylene autosampler vials and then 10 μL will be injected into the HPLC/MS/MS system. The ratio of peak areas of PEth 16:0/18:1 to that of d5-PEth 16:0/18:1 of the unknown samples will be compared against a linear regression of ratios of calibrators from 0 to 4,000 ng/mL.

#### 2.5.2. Urine ethyl-glucuronide (uEtG)

uEtG will be assessed at each visit using a dipcard (≥300 ng/mL threshold, Healgen Scientific). Participants will self-collect urine samples off camera using a point-of-care immunoassay cup that assesses substances other than alcohol (see Section 2.5.4). Participants will conduct the uEtG dipcard test on camera with study staff assistance.

#### 2.5.3. Self-reported alcohol use

Self-reported daily alcohol use will be assessed at each visit using the Alcohol Timeline Followback (ATLFB) [27]. This information will reflect the thirty days prior to the enrollment visit, as well as “since the last visit” at each subsequent visit. Additionally, we will record hours since the last drinking episode and the number of drinks at the last drinking episode.

#### 2.5.4. Alcohol-associated harms

Self-reported cigarette use will be assessed per Timeline Followback, and nicotine dependence assessed using the Fagerstrom Test for Nicotine Dependence. The use of costly medical care (non-study related resources) will also be measured using the Non-study Medical and Other Services (NMOS) form, which captures self-reported healthcare utilization for inpatient, outpatient, and emergency department visits, inpatient, residential, and outpatient treatment days, mental health treatment visits, and substance use disorder medications [30–32]. Lastly, a urine point-of-care immunoassay cup will be used to assess uEtG and cannabis use (tetrahydrocannabinol ≥50 ng/mL; UScreen).

#### 2.5.5. ANA framework

The ANA will serve as our conceptual framework to identify pre-intervention (e.g., executive functioning, incentive salience, negative emotionality) and in-treatment (e.g., alcohol abstinence) factors related to post-treatment outcomes over the 12-month follow-up period [28]. We will use the validated 15-item self-report measure of ANA constructs [29], in addition to administering performance-based measures of executive functioning (i.e., divided attention, working memory, mental flexibility, response inhibition, impulsivity, and delayed discounting). Performance-based measures will be self-administered remotely using the TestMyBrain.org platform [30].

#### 2.5.6. Economic measures

The NMOS form will be used to measure resource utilization needed to inform the state-policymaker and societal perspectives, including criminal-legal, social safety-net, labor/school productivity, travel time to medical care, and caregiver time. This form utilizes time-anchoring methodology to assess participant resource use over the 30 days prior to the enrollment visit, and then “since your last visit” at each subsequent administration. The Drug Abuse Treatment Cost Analysis Program instrument will be used to guide the microcosting analysis (see 3.3.2) [31].

#### 2.5.7. Implementation measures

The percentage of visits missed will serve as an indicator of attrition. Additionally, we will track the frequency of, and reason for, delayed PEth sample shipments. If a sample is shipped 1–7 days after the study visit, instead of the same say as the study visit, it will be coded as “late.” If a sample is not shipped within 7 days, it will be coded as missing, the reinforcers withheld, and reinforcers reset to $20 when the next PEth negative sample is received. To assess participant satisfaction for this study, we will administer CM participants the Client Satisfaction Questionnaire, in which an a priori mean score >23 will indicate overall satisfaction with our telehealth-based CM procedures [32].

### 2.6. Adverse events

Determination of intervention-related adverse events will be made, and procedure modifications executed, if needed. If modifications cannot be made, the study will be terminated. Risks related to suicidality, dangerous alcohol use, and withdrawal symptoms will be assessed at every visit. Withdrawal symptoms will be measured using the Sweating, Hallucinations, Orientation, Tremor (SHOT) scale, with adaptations for virtual evaluation [33]. If a participant scores >3 on the SHOT, we will administer the Clinical Institute Withdrawal Assessment for Alcohol to determine if immediate medical intervention is needed [33].

## 3. Analytic plan

### 3.1. Preliminary analyses

Our data and supporting documents will be submitted to the National Institute on Alcohol Abuse and Alcoholism’s National Data Archive (NDA) and will be available within three years of completion of the study. Groups will be compared for differences in demographics and pre-treatment alcohol use. Means and standard deviations will characterize continuous variables, and percentages and frequencies characterize categorical variables. We will assess the impact of sex as a biological variable. Our alpha threshold for statistical significance will be 0.05.

### 3.2. Treatment efficacy

We will follow an intention-to-treat approach to provide unbiased group comparisons. Treatment efficacy will be assessed per our primary outcome, PEth-defined abstinence. In the Starting Phase, abstinence will be defined as a week-over-week decrease in PEth levels (yes/no). During the Sustaining Phase and Follow-up Phase, abstinence will be defined as PEth <20 ng/mL (yes/no). Separate generalized estimating equations (GEE) with logit link functions will be used to assess abstinence during treatment (11 assessments across 26 weeks) and follow-up (4 assessments across 52 weeks). The frequency of PEth-defined excessive drinking (≥200 ng/mL; yes/no) will also be analyzed with GEE. GEEs with appropriate link functions will also assess additional outcomes for the longest duration of PEth-defined abstinence, uEtG results (positive/negative), self-reported days of drinking, self-reported drinks per day, and alcohol-associated harms (cigarette smoking, drug use, utilization of costly medical care). All GEE models will include effects for groups, biological sex, time, and the group-by-time interaction. Statistically significant group-by-time interactions will be evaluated with Bonferroni corrected post hoc tests.

### 3.3. Additional analyses

#### 3.3.1. ANA variables

GEE with logit link functions for binary outcomes and identity link functions for continuously distributed outcomes will be used to investigate whether ANA variables collected at the enrollment visit are predictive of abstinence across follow-up. Further modeling will include in treatment PEth-defined abstinence and excessive drinking as potential predictors of abstinence in follow-up. For in treatment repeated variables (see Table 1), we will take the average of the repeated assessments as point-estimate predictors.

#### 3.3.2. Economic analyses

Economic analyses will examine resource use from the healthcare sector, state-policymaker, and societal perspectives [34–36]. The healthcare-sector perspective includes medical costs incurred by the system on behalf of participants, including the cost of interventions, and participant out-of-pocket costs. The state-policymaker perspective informs resource allocation decisions made in the interest of the public, who are responsible for funding healthcare for many individuals with AUD, as well as criminal-legal resources, and social safety-net programs. The societal perspective accounts for all resources utilized by, or on behalf of, individuals with AUD, including those mentioned above, and those more broadly associated with untreated or undertreated AUD and comorbid conditions, such as premature mortality, reduced labor productivity, and costs incurred by crime victims [34,37].

First, microcosting analyses will be conducted to identify resource use and estimate the costs associated with implementing and sustaining CM relative to the Control condition. Next, between-arm difference in predicted-mean costs will be calculated according to each stakeholder perspective over the intervention and follow-up periods. The resource costing method will be used to assign national real-world unit costs to each resource utilized. Then, between-arm differences in predicted-mean values for effectiveness measures, alcohol-free years and quality-adjusted life-years (QALY), will be calculated for each time period with generalized linear mixed-models to adjust for potential confounding factors. Finally, incremental cost-effectiveness ratios (ICERs) will be calculated for each perspective/effectiveness measure by time period, and cost-effectiveness acceptability curves estimated to assess the uncertainty surrounding each ICER.

### 3.4. Missing data

Missingness will be handled with maximum likelihood or multiple imputation and other sensitivity analyses, including “missing not at random” approaches [24,38–40]. Maximum likelihood and multiple imputation have both shown exceptional performance compared to other methods of handling missing data when the assumption of “missing at random” can be satisfied [40–44].

### 3.5. Power

Our sample size (*n* = 200) is based on statistical power calculations with the primary outcome of detecting group differences in PEth-assessed alcohol abstinence across 11 assessments during treatment and 4 assessments in follow-up. Our missing data techniques will recapture some power lost due to attrition, but we take a conservative approach to ensure adequate power by basing power calculations on a sample size reduced by 25% (*n* = 150). Our pilot study found an increase in abstinence of 43% in the CM group compared to 0% in the Control group. Using methods previously described [45], this effect would result in >99% power with *n* = 150 when comparing abstinence among groups in treatment (G*Power software). While this effect is promising, CM meta-analyses and large RCTs consistently demonstrate odds ratios (ORs) as effect sizes ranging from 1.7 to 4.5 for increasing substance use abstinence [23,46–51]. In addition, CBT4CBT, which all participants will receive, has an effect size on substance use ranging from *d* = 0.11–0.37. These are modest and consistent with CBT interventions that are commonly paired with CM in past trials where CM demonstrated superiority to CBT [47,49,52]. Based on our pilot study, we expect an effect size no smaller than OR = 1.7, which accounts for the underlying effect of CBT in both groups. In the treatment and follow-up period, at a sample size of *n* = 150, we will have at least 80% power to detect a modest effect size of OR = 1.34.

For the secondary aim of reducing the frequency of PEth-defined excessive drinking, our group has consistently found secondary improvements in the odds of substance-related harms to be OR = 1.80 or larger. Assuming a sample size of n = 150, we estimate at least 80% power to detect an OR = 1.80 for reduction in PEth-defined excessive drinking during treatment or the follow-up period.

For analyses of the ANA, our proposed sample size of n = 150 will provide at least 80% power to identify an OR = 1.44 when there are as many as 10 predictors being modeled. This OR corresponds to a small but clinically meaningful change predicted by demographics, our ANA variables, and in-treatment PEth-defined abstinence.

Power for our economic analysis is based on assumptions regarding maximum willingness-to-pay value [53,54]. Using varying estimates from previous relevant RCTs conducted over similar time frames, we estimate at least 80% power that the intervention is a good value at the recommended willingness-to-pay threshold of $150,000 per QALY [55].

## 4. Discussion

This randomized controlled clinical trial assesses the efficacy of a telehealth-based CM intervention for AUD in a large national sample. Telehealth delivery has some potential limitations, such as access to and comfort with technology, but advantages, like reduced stigma and travel, likely outweigh the disadvantages. For instance, 64% of participants in our telehealth-based CM pilot study were treatment-naïve [7], suggesting that telehealth interventions might be a low-burden and low-barrier treatment option for individuals with AUD.

While CM is an evidence-based treatment for AUD and a powerful tool for initiating abstinence, evidence for sustained post-intervention effects is less conclusive [23,47,48,52,56]. In efforts to address this gap in CM research, the follow-up period for this study is 12 months in duration compared to 6 months in traditional CM models. Another strength of this study is the utilization of the ANA framework and alcohol biomarkers to identify potential predictors of treatment response and long-term outcomes. These predictors could provide opportunities to improve treatment efficacy and better direct care for this population (e.g., individualized treatment approaches). Lastly, this study will fill a critical gap in the CM literature by providing essential information for providers regarding resource and cost requirements for implementing this telehealth-based CM model.

A limitation of the study is that there is up to a one-week delay between submission of blood samples and provision of CM reinforcers. If we do not observe an effect of CM on outcomes, it could be a result of this delay. However, in our pilot study, CM participants did not report concerns regarding the week delay (i.e., surveys and qualitative interview) and achieved levels of alcohol abstinence consistent with previous CM studies [7]. Another potential limitation is the increased burden for the CM group, depending on how long participants remain in the Starting Phase. More visits might contribute to higher attrition in the CM condition, or it could lead to lower levels of attrition because individuals who are unable to achieve long-term abstinence (i.e., <20 ng/mL) will have more frequent visits and a lower bar for achieving reinforcers. The effects of an adaptive reinforcement schedule on attrition rates in the CM group will be assessed.

## 5. Conclusion

The primary goal of this clinical randomized controlled trial is to develop a treatment approach that removes barriers to interventions for individuals with AUD. We propose that using PEth to objectively assess drinking during a telehealth CM intervention will meet this goal by reducing the frequency of assessments and eliminating the need for inperson visits. The comprehensive cost-effectiveness analysis will also inform whether CM is viable from healthcare-sector, state-policymaker, and societal perspectives. If our telehealth-based CM intervention reduces alcohol use and is cost-effective, it could be used to provide effective and low barrier treatment approach for the millions of individuals with an AUD who do not receive in-person care.

## Figures and Tables

**Table 1:** Study measures and data collection schedule: See methods for a description of each measure. Acronyms included are phosphatidylethanol (PEth), urine ethyl glucuronide (uEtG), Non-study Medical and Other Services (NMOS), Drug Abuse Treatment Cost Analysis Program (DATCAP), Client Satisfaction Questionnaire (CSQ-8), and the Sweating, Hallucinations, Orientation, Tremor scale (SHOT).

Measure	Enrolment Visit	Every Visit	Treatment Visits Week 4, 8, 12, 16, 20, 26	Follow-Up Visits Week 30, 38, 52, 78
Eligibility Criteria	✓			
Demographics	✓			
PEth-Defined Abstinence <20 ng/mL	✓	✓		
PEth-Defined Excessive Drinking ≥ 200 ng/mL	✓	✓		
uEtG-Defined Recent Drinking: ≥ 300 ng/mL	✓	✓		
Self-Reported Alcohol Timeline Followback	✓	✓		
Self-Reported Cigarette Timeline Followback	✓	✓		
Nicotine dependence: Fagerstrom	✓		✓	✓
Drug Use: 5-Panel Cup	✓	✓		
ANA Model: Questionnaire	✓	✓		
ANA Model: TestMyBrain	✓			
Healthcare Utilization: NMOS	✓		✓	✓
Intervention cost: DATCAP	✓			
Attrition: Percent missed visits		✓		
Client Satisfaction: CSQ-8			Weeks 4, 26	
Alcohol withdrawal: SHOT	✓	✓		
Adverse Events	✓	✓		

## Data Availability

No data was used for the research described in the article.
